# Identification of the Host Substratome of *Leishmania*-Secreted Casein Kinase 1 Using a SILAC-Based Quantitative Mass Spectrometry Assay

**DOI:** 10.3389/fcell.2021.800098

**Published:** 2022-01-03

**Authors:** Despina Smirlis, Florent Dingli, Valentin Sabatet, Aileen Roth, Uwe Knippschild, Damarys Loew, Gerald F. Späth, Najma Rachidi

**Affiliations:** ^1^ Institut Pasteur, Université de Paris, Institut National de Santé et Recherche Médicale INSERM U1201, Unité de parasitologie Moléculaire et Signalisation, Paris, France; ^2^ Hellenic Pasteur Institute, Athens, Greece; ^3^ Laboratoire de Spectrométrie de Masse Protéomique (LSMP), Centre de Recherche, Institut Curie, PSL Research University, Paris, France; ^4^ Department of General and Visceral Surgery, Centre of Surgery, University Hospital Ulm, Ulm, Germany

**Keywords:** Casein kinase I, Substrate screen, Leishmania, Host-pathogen interactions, Cancer, SARS-CoV2

## Abstract

Leishmaniasis is a severe public health problem, caused by the protozoan *Leishmania*. This parasite has two developmental forms, extracellular promastigote in the insect vector and intracellular amastigote in the mammalian host where it resides inside the phagolysosome of macrophages. Little is known about the virulence factors that regulate host-pathogen interactions and particularly host signalling subversion. All the proteomes of *Leishmania* extracellular vesicles identified the presence of *Leishmania* casein kinase 1 (L-CK1.2), a signalling kinase. L-CK1.2 is essential for parasite survival and thus might be essential for host subversion. To get insights into the functions of L-CK1.2 in the macrophage, the systematic identification of its host substrates is crucial, we thus developed an easy method to identify substrates, combining phosphatase treatment, *in vitro* kinase assay and Stable Isotope Labelling with Amino acids in Cell (SILAC) culture-based mass spectrometry. Implementing this approach, we identified 225 host substrates as well as a potential novel phosphorylation motif for CK1. We confirmed experimentally the enrichment of our substratome in bona fide L-CK1.2 substrates and showed they were also phosphorylated by human CK1δ. L-CK1.2 substratome is enriched in biological processes such as “viral and symbiotic interaction,” “actin cytoskeleton organisation” and “apoptosis,” which are consistent with the host pathways modified by *Leishmania* upon infection, suggesting that L-CK1.2 might be the missing link. Overall, our results generate important mechanistic insights into the signalling of host subversion by these parasites and other microbial pathogens adapted for intracellular survival.

## Introduction

*Leishmania* causes immuno-pathological diseases including cutaneous, muco-cutaneous, and visceral leishmaniasis, leading to severe morbidity and mortality. This parasite has two developmental stages, in the insect vector as an extracellular promastigote form, and in the mammalian host as an intracellular amastigote form where it resides inside the phagolysosome of macrophages. Our understanding of host-*Leishmania* interactions was very limited until the discovery of the *Leishmania* exo-proteome, revealing potential mechanisms by which the parasite subverts its host cell (Silverman et al., 2010) (Atayde et al., 2015; Rachidi et al., 2021). Among the proteins released by *Leishmania*, casein kinase 1.2 (LmjF35.1010, L-CK1.2) is particularly remarkable. Indeed CK1 family members as signalling kinases are involved in the regulation of multiple processes, such as apoptosis or cell cycle. Moreover, manipulation of the host cell CK1-signalling pathways is common to many intracellular pathogens, from viruses to eukaryote pathogens (Jayaswal et al., 2010; Xia et al., 2018; Rachidi et al., 2021; Dorin-Semblat et al., 2015), suggesting that the release of L-CK1.2 might be essential for macrophage subversion by *Leishmania*. Similarly to its human orthologs, *Leishmania* CK1.2 has a ubiquitous distribution in the parasite and is detected in the flagellum, flagellar pocket, the cytoplasm, or strongly associated with the cytoskeleton (Martel et al., 2020). The localisation of L-CK1.2, but not its activity requires its C-terminus domain (Martel et al., 2020), which might mostly mediate the interaction of L-CK1.2 to interacting partners (Knippschild et al., 2014). L-CK1.2 was shown to be part of the core cargo of exosomal proteins (Silverman et al., 2011; Silverman et al., 2010), present in exosomes released by promastigotes in the insect vector (Atayde et al., 2015) and enriched in exosomes released by amphotericin B-resistant parasites but not in that released by miltefosine- or antimony-resistant parasites (Douanne et al., 2020; Rachidi et al., 2021), suggesting that it has an important role for parasite survival in the insect and mammalian hosts. We and others have shown that L-CK1.2 is essential for intracellular parasite survival (Rachidi et al., 2014; Baker et al., 2021) and could be evolutionary selected for its capacity to interact with and phosphorylate host proteins to modulate macrophage biological and immune processes (Rachidi et al., 2021; Rachidi et al., 2014). Indeed, L-CK1.2 phosphorylates the human IFNAR1 receptor, which leads to the attenuation of the cellular response to interferon α/β (Liu et al., 2009). Altogether, these findings are consistent with L-CK1.2 being a master regulator of *Leishmania* intracellular survival. To determine its contribution to the regulation of host-pathogen interactions, identification of the host pathways it regulates through the systematic identification of substrates is crucial. However, the low stoichiometry of protein phosphorylation, the presence of endogenous kinases as well as the reversibility of the phosphorylation by phosphatases render systematic mapping of the cellular substratome extremely challenging. This is particularly true when handling pleiotropic signalling kinases such as CK1 family members, able to phosphorylate hundreds of substrates (Knippschild et al., 2014). We thus developed a technology, easily applicable to other protein kinases, that allows efficient identification of substrates. Applying this pipeline on L-CK1.2, we identified 225 host substrates that shed important new lights on parasite immune and biological subversion of its host. Furthermore, we validated our approach, which might become a powerful new tool to study mechanisms of host-pathogen interactions and might provide host targets for host-directed therapy against Leishmaniasis.

## Material and Methods

### SILAC Labelling and Lysate Preparation

For labelling cells by SILAC, equal numbers of THP-1 monocytes (2 × 10^5^ ml^−1^) were seeded in RPMI 1640 without Lysine and Arginine (Thermo Fisher Scientific), supplemented either with natural amino acids (L-Lysine, 0.274 mM; L-Lysine, 1.15 mM; Arginine, 1.15 mM) or with the same concentrations of amino acid isotopes 2H4-Lysine (Lys4) and 13C6--Arginine (Arg6) (Thermo Scientific). The medium was supplemented with 50 μM β-mercaptoethanol, 50U mL^−1^ penicillin, 50 μgmL^−1^ streptomycin and 10% (v/v) of dialysed Fetal Bovine Serum (Sigma). Cells were split and seeded before reaching a concentration of 10^6^ ml^−1^ in fully supplemented SILAC medium, for a period of at least 15 days 0.75 to 1 × 10^8^ cells were then differentiated for 48 h into macrophages by the addition of 10 ng ml^−1^ PMA. Cells cultivated in SILAC medium were washed three times in PBS and lysed in RIPA lysis and extraction buffer (Thermo Scientific) containing one tablet per 10 ml of cOmplete™ protease Inhibitor Cocktail tablets (Sigma). Cell extracts were incubated on ice 30 min, sonicated 5 min, and centrifuged 15 min at 14,000 g to eliminate cell debris. Proteins were quantified in the supernatants using the RC DC™ protein assay kit (Bio-Rad), according to the manufacturer’s instructions. For free ATP depletion, protein extracts were dialyzed overnight at 4°C in 1 L of dialysis solution (1× PBS, 1 mM EDTA, 1 mM dithiothreitol) in a Slide-A-Lyzer dialysis cassette (Pierce).

### Expression and Purification of L-CK1.2 and L-CK1.2-K40A

Bacterial expression plasmid for L-CK1.2 was generated as previously described (Rachidi et al., 2014). Bacterial expression plasmid carrying L-CK1.2-K40A (kinase dead) was generated by site-directed mutagenesis as previously reported (Rachidi et al., 2014). Recombinant proteins were induced in Rosetta™ (DE3) Competent Cells (Novagen) with 0.02% (w/v) L-arabinose for 3 h at 25°C. Cells were harvested and resuspended in lysis buffer as previously described (Rachidi et al., 2014). Briefly recombinant kinases were purified on co-nitrilotriacetic acid agarose (Pierce) and eluted in 300 mM imidazole in PBS containing 60 mM β-glycerophosphate, 1 mM sodium vanadate, 1 mM sodium fluoride and 1 mM disodium phenylphosphate. Protein eluates were supplemented with 15% glycerol and stored at −80°C.

### Expression and Purification of Human GST-CK1δ^TV1^

Bacterial expression of GST-CK1δ^TV1^ was induced with 0.5 mM IPTG at an OD_600_ of 0.6 AU in *E. coli* SoluBL21^TM^ (Genlantis) and incubated for 18 h at 18°C. Cells were harvested and resuspended in lysis buffer containing 20 mM Tris-HCl (pH 7.6), 150 mM NaCl, 0.5% NP-40, 10% glycerol, 1 mM EDTA, 1 mM EGTA, 1 mM benzamidzine, 0.25 μg/ml aprotinin and 1 mM DTT. The lysate was centrifuged at 15,000 × g for 30 min. The cleared lysate was incubated with 300 µL of glutathione sepharose suspension (50% Glutathione Sepharose® 4 Fast Flow (Cytiva) in PBS) for 2 h at 4°C. Afterwards, glutathione sepharose beads were washed three times in lysis buffer containing 300 mM NaCl and twice in washing buffer (20 mM Tris-HCl (pH 7.6), 50 mM NaCl, 0.25 μg/μL aprotinin, 1 mM EDTA). The bound proteins were eluted with 1 ml elution buffer containing 50 mM Tris-HCl (pH 7.6), 1 mM EDTA and 5 mM reduced glutathione. Proteins were stored in 10% glycerol at −80°C after shock freezing in liquid nitrogen.

### Protein Kinase Assay and Phosphatase Treatment

For phosphatase treatment, 500 μg of “heavy” or “light” THP-1 protein extract were dephosphorylated with 50 U of Antarctic phosphatase (NEB) in Antarctic phosphatase buffer (NEB) for 30 min at 37°C. Phosphatase activity was heat inactivated at 65°C for 15 min. The kinase assay for the “heavy” or “light” protein extracts (500 μg) were performed in buffer C (60 mM β-glycerophosphate, 30 mM *p*-nitrophenyl phosphate, 25 mM MOPS [morpholinepropanesulfonic acid], 5 mM EGTA, 15 mM MgCl_2_, 1 mM dithiothreitol, 0.1 mM sodium vanadate; pH 7.0) in the presence of 15 μM ATP and 200 ng of recombinant L-CK1.2 or kinase dead L-CK1.2-K40A. The reaction was performed in triplicate at 30°C for 45 min. For mock reactions no kinases were added. Reactions were stopped with the addition of 10 μM of D4476 {4- [4-(2,3-dihydro- 1,4- benzodioxin-6-yl)- 5- 2-pyridinyl)- 1*H*- imidazol-2-yl]} benzamide, a specific inhibitor of CK1 known to inhibit *L-CK1.2* activity (Rachidi et al., 2014), followed by heat inactivation at 65°C for 15 min. Next, kinase inactivation heavy and light protein samples were mixed in a 1:1 ratio, and precipitated in 80 (v/v) % ice-cold acetone and stored at −80°C prior to LC/MS-MS analysis. For IVKA with L-CK1.2, 15 μg of total THP-1 protein extracts or 0.5–1.0 μg of recombinant proteins, GST-14-3-3γ (Enzo #BML-SE313-0100), RCN2-6XHis (Abcam #ab125644) Glo1 (Abcam # ab206792), SNAP23 (Abnova #H00008773-P01) and TAF7 (Abnova #H00006879-P01), were assayed with 0.2 μg of L-CK1.2 or L-CK1.2-K40A, as described in Rachidi et al. (2014). Incorporated ^32^P was monitored by SDS-PAGE and autoradiography. For IVKA with human GST-CK1δ^TV1^, 6 ng of kinase was incubated with the 0.5–1.0 μg of recombinant proteins (see above) in 1x kinase buffer (250 mM Tris-HCl (pH 7.0), 100 mM MgCl_2,_ 1 mM EDTA) and 15μΜ ^32^P-γATP in 30 μL final volume for 30 min at 30°C. All kinase assays were performed at least three times.

### Phospho-Peptide Enrichment

Phosphorylated peptides were enriched using Titansphere^TM^ Phos-TiO kit centrifuge columns (3 mg/200 µL, cat. No. 5010-21312, GL Sciences), as described by the manufacturer. After elution from the Spin tips, phospho-peptides were vacuum concentrated to dryness and reconstituted in 0.1% formic acid. Samples were then loaded onto a custom-made C18 StageTips packed by stacking one AttractSPE® disk (#SPE-Disks-Bio-C18-100.47.20 Affinisep) and 2 mg beads (#186004521 SepPak C18 Cartridge Waters) into a 200 µL micropipette tip for desalting. Enriched phospho-peptides were eluted using 40/60 MeCN/H2O + 0.1% formic acid and vacuum concentrated to dryness.

### Liquid Chromatography-Tandem Mass Spectrometry

LC was performed with an RSLC nano system (Ultimate 3000, Thermo Scientific) coupled online to an Orbitrap Fusion Tribrid mass spectrometer (Thermo Scientific). Peptides were first trapped on a C18 column (75 μm inner diameter × 2 cm; nanoViper Acclaim PepMap^TM^ 100, Thermo Scientific) with buffer A (2/98 MeCN/H2O in 0.1% formic acid) at a flow rate of 2.5 μL/min over 4 min. Separation was then performed on a 50 cm × 75 μm C18 column (nanoViper Acclaim PepMap^TM^ RSLC, 2 μm, 100Å, Thermo Scientific) regulated to a temperature of 55°C with a linear gradient of 2–25% buffer B (100% MeCN in 0.1% formic acid) at a flow rate of 350 nL/min over 211 min. Separation of phospho-peptide samples without phosphatase was performed with buffer A’ (5/98 DMSO/H2O in 0.1% formic acid) and B’ (5/95 DMSO/MeCN in 0.1% formic acid) by using a linear gradient of 2–30% with the same time and flow rate as previous gradient. Full-scan MS was acquired in the Orbitrap analyzer with a resolution set to 240,000, a mass range of m/z 400–1500 and a 4 × 10^5^ ion count target. ions from each full scan were isolated and futher HCD fragmented with normalized collision energy of 30% and rapid scan MS analysed in the linear ion trap. The MS^2^ ion count target was set to 2 × 10^4^ and only those precursors with charge state from 2 to 7 were sampled for MS^2^ acquisition.

### Data Analysis

For identification, the data were searched against the *Homo sapiens* (UP000005640) UniProt database using Sequest-HT through Proteome Discoverer (PD, version 2.4). Enzyme specificity was set to trypsin and a maximum of two-missed cleavage sites was allowed. Oxidized methionine, Met-loss, Met-loss-Acetyl, Ser/Thr/Tyr phosphorylation, N-terminal acetylation, heavy ^2^H_4_-Lysine (Lys4) and ^13^C_6_-Arginine (Arg6) were set as variable modifications. Carbamidomethyl of cysteines were set as fixed modification. Maximum allowed mass deviation was set to 10 ppm for monoisotopic precursor ions and 0.6 Da for MS/MS peaks. The resulting files were further processed using myProMS v3.9.2 (Poullet et al., 2007). The Sequest-HT target and decoy search result were validated at 1% false discovery rate (FDR) with Percolator at the peptide level. Technical replicates (*n* = 3) were merged using the MSF files node and a SILAC-based phospho-peptides quantification was performed by computing peptides XICs (Extracted Ion Chromatograms). The phosphosite localization accuracy was estimated by using the PtmRS node in PD (version 2.4), in PhosphoRS mode only. Phosphosites with a localization site probability greater than 75% and with at least two SILAC measurements per peptide were quantified at the peptide level. Mass spectrometry proteomics data have been deposited to the ProteomeXchange Consortium via the PRIDE (Perez-Riverol et al., 2019) partner repository with the dataset identifier PXD026220.

### Motif Discovery

Unique phospho-peptides sequences, matching the strict selection criteria, were aligned on phosphorylated serine and threonine with 5 flanking amino acids. PhosphoSitePlus phosphosite plus [https://www.phosphosite.org/homeAction.action (Hornbeck et al., 2015)] was used to compute motif analysis enrichment (automatic background selection; significance of 1e-6; support threshold of 0.1) and to generate corresponding sequence logo (automatic background selection; frequency change algorithm (Vacic et al., 2006).

### STRING Network Visualization and Gene Ontology Enrichment Analysis

The dataset was analyzed for protein-protein interactions and visualized using the STRING plugin [string, https://string-db.org/ (Szklarczyk et al., 2019)] of the Cytoscape software package [version 3.8.2, https://cytoscape.org/ (Shannon et al., 2003)]. Each node represents a substrate and each edge represents a protein-protein interaction. Functional enrichment analysis of the dataset was performed using the g:profiler web server (https://biit.cs.ut.ee/gprofiler/gost) and the following criteria: only annotated genes, with a significance threshold of 0.05, select GO terms of less than 5,000 genes, and only focusing on GO “biological process.” Results were visualized using the EnrichmentMap plugin of the Cytoscape software package [version 3.3, http://apps.cytoscape.org/apps/enrichmentmap (Merico et al., 2010)], with a p-value and a Q-value above 0.05 and an edge cut-off of 0.375. Node color represents the enrichment p-value (see legend Figure 4). Node size is proportional to the total number of genes belonging to the corresponding gene-set. The edge corresponds to the Annotation shared between two nodes, with edge thickness corresponding to the number of shared genes. Node clusters were identified and annotated by the AutoAnnotate plugin of cytoscape (version 1.3.3 https://autoannotate.readthedocs.io/en/latest/).

## Results

### Strategy and Establishment of the Experimental Protocol

**FIGURE 1:**
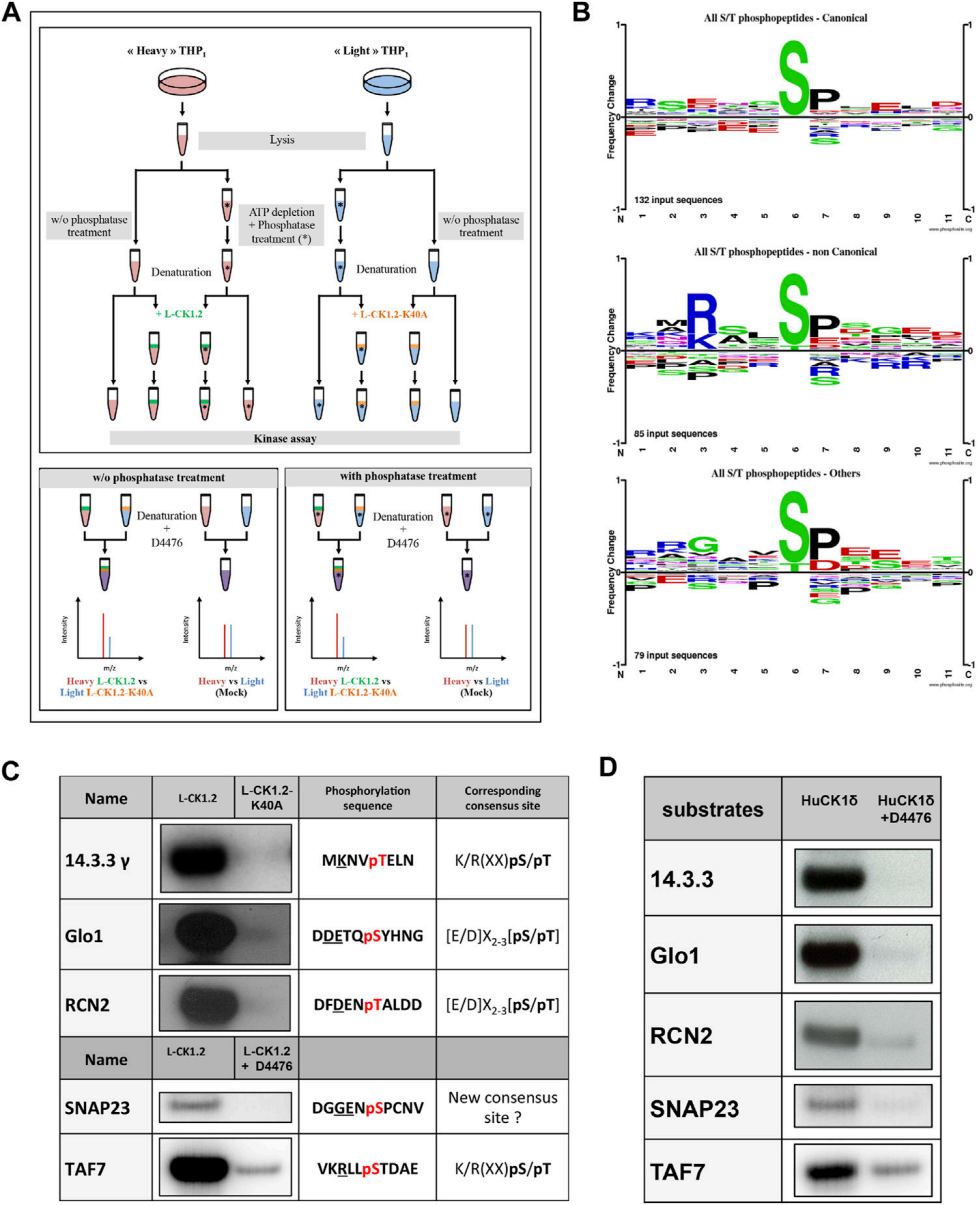
Experimental workflow and validation of substrate dataset. **(A)** Upper Panel. Workflow diagram showing the experimental strategy used to reveal L-CK1.2 substratome derived from THP-1 lysates. THP1 cells were cultured and differentiated in the presence of natural amino acids (light, blue) or stable amino acid isotopes (heavy, red). Equal amounts per reaction (0.5 mg) of heavy or light lysates were treated with phosphatase and ATP depleted (*) or not and denatured by heat inactivation to remove endogenous kinase activities. The phosphatase reactions were stopped by heat inactivation. Lysates were then subjected to IVKA in presence of recombinant L-CK1.2 (green), L-CK1.2-K40A (kinase-dead, orange), or were mock treated with equal amounts of kinase elution buffer, in triplicate. The reactions were stopped with heat inactivation and addition of 10 μM D4476. Lower panel. Equal amounts (0.5 mg) of heavy (L-CK1.2) and light (L-CK1.2-K40A) samples were mixed. In addition, mock heavy and light samples were mixed in a 1:1 ratio and used as a control. The four samples were reduced, alkylated and digested and the resulting phospho-peptides were enriched by TiO2-affinity chromatography, and processed by LC-MS/MS analysis on an Orbitrap fusion mass spectrometer. **(B)** Sequence logos analysis of unique phospho-sites (five amino acids before and after the phosphorylation residues) matching strict selection criteria. Upper panel, for canonical consensus sites; middle panel, for non-canonical sites; and lower panel, for others. The amino acids are labelled according to their chemical properties: green for polar amino acids (G, S, T, Y, C, Q, N), blue for basic amino acids (K, R, H), red for acidic amino acids (D, E), and black for hydrophobic amino acids (A, V, L, I, P, W, F, M). **(C)** Autoradiogram representing IVKA using selected recombinant human proteins and inactive L-CK1.2-K40A (kinase-dead), active L-CK1.2 alone or in presence of the CK1-specific small molecule inhibitor D4476 (10 μM). **(D)** Autoradiogram representing IVKA using selected recombinant human proteins and recombinant CK1δ^TV1^ alone or in the presence of D4476.

To identify the L-CK1.2 host-substratome, we implemented an experimental workflow designed to quantify TiO_2_-enriched phospho-peptide stoichiometry by LC-MS/MS in metabolically-labelled, heat inactivated THP-1 macrophage protein lysates after their phosphorylation by recombinant L-CK1.2 (Figure 1A). In order to increase the number of sites available for *de novo* L-CK1.2 phosphorylation prior *in vitro* kinase assay (IVKA), we depleted the lysates in ATP before treating them with Antarctic phosphatase. However, since CK1 recognises, as a main consensus site, the following motif [S/T]X_2-3_ [**pS**/**pT**], where the second S/T residue is only accessible for CK1 phosphorylation if the first S/T residue is primed (a.k.a. phosphorylated) by other kinases, we also performed an experiment without phosphatase treatment. TiO_2_-enriched phospho-peptide stoichiometry was calculated from technical triplicate as a ratio of Heavy_L-CK1.2_/Light_L-CK1.2-K40A_ and comparisons were made to phospho-peptide ratios of Heavy/Light mock reactions without kinases.

For establishing appropriate experimental conditions, several pilot experiments were carried out. To decrease the background activity of endogenous kinases, the lysate of THP-1 macrophages was pulse-heated to denature endogenous kinases (Supplementary Figure S1). Denaturation efficiency was demonstrated by the absence of ^32^P incorporation in the background control (Supplementary Figure S1, lane 2). In contrast, the *de novo* phosphorylation of denatured THP-1 proteins by active L-CK1.2 was detected, showing that L-CK1.2 phosphorylates proteins present in the macrophage lysate (Supplementary Figure S1, lane 1). To increase the number of sites available for *de novo* phosphorylation in the macrophage lysate, two steps were added to the pipeline (Figure 1A). The protein lysates were dephosphorylated by Antarctic phosphatase and depleted of free ATP by dialysis to prevent any additional phosphorylation. Similar patterns of total protein staining (Supplementary Figure S1, right panel) and phosphorylation profile (Supplementary Figure S1, left panel) were observed between samples treated or not with phosphatase and thus dialysed or not, suggesting that protein degradation following dialysis was minimal under our experimental conditions. The increase of ^32^P incorporation into substrates following phosphatase treatment (Supplementary Figure S1, lane 3) confirms that previously many sites were inaccessible to *de novo* phosphorylation. To reduce technical errors during phospho-peptides enrichment, to limit missing values and to perform quantitative analyses, we used Stable Isotope Labelling with Amino acids in Cell culture (SILAC). This method relies on the metabolic incorporation of either “Heavy” [^2^H_4_-Lysine (Lys^4^) and ^13^C_6_-Arginine (Arg^6^)] or natural (“Light”) [lysine (Lys^0^) and arginine (Arg^0^)] amino acids (Ong et al., 2002; Ong and Mann, 2006; Basken et al., 2018). We validated the percentage of Lys4 incorporation in THP-1 macrophage proteins by LC-MS/MS analysis, which was over 99%. Finally, to reduce the risk of selecting false positive signals, we performed mock kinase assays using the “light” and “heavy” macrophage lysates without adding the kinases, to discard sites already differentially phosphorylated prior to the kinase assays.

### Identification of L-CK1.2 Host Cell Substrates

Three independent reactions for each condition were carried out, and two independent protein extracts treated or not with phosphatase were used. In the absence of phosphatase treatment, 7,752 unique enriched phospho-peptides belonging to 3,544 unique proteins were identified of which 65% were quantified (see Supplementary Figures S2A, B for Venn diagrams). The same analysis was performed with samples pre-treated with phosphatase, and 15,852 enriched phospho-peptides (5,158 proteins) were identified of which 66% were quantified (Supplementary Figures S2C, D), demonstrating the efficiency of the phosphatase treatment. However, the increase in phospho-peptides also in the H/L control (Supplementary Figures S2A, B) suggests that the pulse-heating step did not completely abrogate but only reduced the activity of endogenous kinases to levels undetectable by autoradiography (Supplementary Figure S1).

To determine *L-CK1.2* substrates, the following selection criteria were applied: 1) A probability of correct localisation of the phosphorylation site on validated peptides greater than 75%, as calculated by PtmRS software (see Data analysis of Material and methods), 2) the ratio Heavy_L-CK1.2_/Light_L-CK1.2-K40A_ equal or above two to select the sites phosphorylated by L-CK1.2 (Sugiyama et al., 2019); and/or 3) the ratio Heavy_L-CK1.2_/Light_L-CK1.2-K40A_ at least twofold higher than that of the control Heavy/Light to only select the newly phosphorylated sites. As we detected a high number of unique peptides identified in the presence of active kinase and not in that of inactive kinase, p-value could not be used, as statistical analyses either lacked power or were not available depending if a unique phosphopeptide was identified in one or more replicates of one state, respectively. We chose not to use data imputation to force statistical analyses, as current methods are not really suitable for phospho-proteomics, and they often add noise. We chose a stringent fold-change cut-off of 2, similar to the approach of Sugiyama et al., 2019 using for the discovery of the substrates of human kinases (Sugiyama et al., 2019). In addition, to strengthen our selection process, and considering that the overlap between replicates is about 25% at best, we considered reproducibility between replicates and we validated phosphopeptides solely if criteria 1), 2) and 3) were validated at least in two out of three replicates.

Using the above criteria, 99 phospho-peptides (81 proteins) and 158 phospho-peptides (144 proteins) were selected as potential substrates of *L-CK1.2* in absence or in presence of phosphatase, respectively (Table 1). Noticeably, only proteins expressed in non-infected macrophages could be detected in our experimental setting. We missed potential substrates exclusively expressed in infected macrophages. Only fifteen proteins were common to the two datasets. Although consistent with the variability of the data dependent acquisition (DDA) LC-MS/MS analysis, this finding suggests that the phosphatase treatment greatly improves the access of L-CK1.2 to new substrates for which it has more affinity.

**TABLE 1:** Phospho-peptides.

	W/o phosphatase treatment	With phosphatase treatment	Total phospho-sites	%
Total phospho-peptides	99	158	257	
Consensus site for CK1				
Canonical sites	42	84	126	72
Non-canonical sites	31	43	74	
Others sites	31	46	77	28
Sites phosphorylated by huCK1s				
Site phosphorylated *in vitro*	14	9	19	7
Sites detected in human cell phospho-proteomes				
Site phosphorylated *in vivo*	92	149	241	94
SARS-coV2 infection				
Sites phosphorylated during SARS-coV2 infection	20	28	52	20
Tumorigenesis				
Site phosphorylated in cancer cells	83	146	229	89
Sites mutated during tumorigenesis	10	16	26	10

### Validation of the Substratome

Because our approach is *in vitro*, we first checked whether the sites we identified are phosphorylated *in vivo*. To this end, we compared our dataset with existing human phosphoproteomes (Table 1 and Supplementary Table S1, https://www.phosphosite.org/homeAction.action). We found that 94% of the 257 phosphosites were phosphorylated *in vivo* suggesting they are physiologically relevant and accessible *in vivo*. Next, three levels of validation were used to demonstrate that the dataset containing 225 proteins are *bona fide* host *L-CK1.2* substrates (Table 2). First, we showed that our dataset contains 55% of known mammalian CK1 substrates or interacting partners: 11 known substrates, including the interferon-alpha/beta receptor alpha chain (IFNAR1/2) (Liu et al., 2009), Sprouty 2 (SPRY2) (Yim et al., 2015) and Fam83H (Fulcher et al., 2018); 46 potential new substrates obtained by Sugiyama *et al.* using a similar method (Supplementary Table S2) (Sugiyama et al., 2019); as well as 66 known interacting partners, which thus might also be substrates (Buljan et al., 2020). Second, the phospho-peptides, we identified, were highly enriched in known CK1 consensus sites (Xu et al., 2019). Hundred and twenty-six sites display the canonical CK1-phosphorylation motif, [S/T]X_2-3_ [**pS**/**pT**] or **[**E/D]X_2-3_ [**pS**/**pT**] (Table 1; Figure 1B top panel), consistent with the affinity of CK1 for these two motifs (Xu et al., 2019). Seventy-four sites display the non-canonical consensus SLS-Xn-(E/D)n (Ha et al., 2004; Marin et al., 2002; Marin et al., 2003; Marin et al., 1994) or resemble to K/R(X)K/R (XX)**pS**/**pT**, a CK1 consensus site identified in cholesterol and sulfatide binding proteins (Table 1; Figure 1B middle panel) (Kawakami et al., 2008). Surprisingly, this motif was present in only 10 phospho-peptides while the remaining 58 peptides contained a shorter version, K/R (XX)**pS**/**pT**. Finally, seventy-seven phospho-sites did not contain any known CK1 motif (others, Table 1), and might represent novel CK1 phosphorylation motifs (Table 1). We searched for motif enrichment using PhosphoSitePlus and identified [G]XX [**pS**] in 20 phospho-peptides with a p-value of 8.02E-11 (Figure 1B bottom panel and Table 3) and variants of this site: [G]XX [**pS**]XX [E] (7 sites) and [G]XX [**pS**]P (10 sites). Noticeably, all the motifs generated for L-CK1.2 in this study highlight the presence of a proline residue adjacent to the phosphorylated S or T (Figure 1B, position 7), which has not been described for human CK1 motifs (Sugiyama et al., 2019). It might reflect the specificity in the substrate recognition motif of L-CK1.2. Third, we performed an *in vitro* kinase assay using recombinant L-CK1.2 to experimentally validate our substratome data. We included five proteins that might be important for *Leishmania* intracellular survival based on their potential to modulate macrophage functions and/or inflammation. The selected recombinant proteins, namely reticulocalbin 2 (RCN2, Q14257), 14-3-3γ (YWHAG, P61981), lactoylglutathione lyase 1 (Glo1, Q04760), synaptosomal-associated prot 23 (SNAP23, O00161) and transcription initiation factor TFIID subunit 7 (TAF7, Q15545) were subjected to a kinase assay using inactive L-CK1.2-K40A (kinase-dead), active L-CK1.2 alone or in presence of the CK1-specific small molecule inhibitor D4476 (Rachidi et al., 2014). All recombinant proteins were phosphorylated by L-CK1.2, but no phosphorylation was observed in the D4476 and L-CK1.2-K40A controls (Figure 1C). We performed a similar experiment using recombinant human CK1δ and showed that the five substrates were also phosphorylated by the human kinase, confirming the relevance of our substratome for human CK1s (Figure 1D). Altogether, these results confirm that our dataset identified bona fide L-CK1.2 host cell substrates, which validate our approach. Furthermore, these substrates are also targeted by human CK1δ, which is consistent with data obtained by Sugiyama *et al.* showing that 24 L-CK1.2 phospho-sites are also targeted by human CK1s (Table 4).

**TABLE 2:** Proteins.

		W/o phosphatase treatment	With phosphatase treatment	Total proteins	%
Total proteins		81	144	225	—
Known human CK1 substrates or binding partners					
Substrates		32	30	57	55
Binding partners		19	47	66	
*Leishmania* infection					
Proteins differentially regulated during infection		25	47	72	60
Transcripts differentially regulated during infection		20	48	68	
Tumorigenesis					
Prognosis markers	Favourable	17	23	40	76
	Unfavourable	34	57	91	
	Both depending on the cancer type	12	28	40	
SARS-coV2 infection					
Proteins phosphorylated during infection		37	78	115	51

**TABLE 3:** Motifs identified in category “others.”

Motif	ZScore	p-value	Foreground matches	Foreground size
…G…sP…	8.64	0.00E+00	10	69
…G….s…E	8.24	0.00E+00	7	69
…G…s…	6.4	8.02E-11	20	69
…sP…	9.94	0.00E+00	34	69
…sD.E	8.43	0.00E+00	8	69
…sD…	8.73	0.00E+00	11	69
….s…E	5.03	2.43E-07	16	69

**TABLE 4:** Residues also phosphorylated by huCK1s.

Substrate/Uniprot ID	Uniprot	huCK1 paralog	Phosphoryated by huCK1s and L-CK1.2	Protein description
CLIC1_HUMAN	O00299	CK1e	S221	Chloride intracellular channel protein 1
ARC1B_HUMAN	O15143	CK1d	S311	Actin-related protein 2/3 complex subunit 1B
ANXA2_HUMAN	P07355	CK1d/e/g1/g2/g3	S12/S184	Annexin A2
GRP78_HUMAN	P11021	CK1d/g2	S311	78 kDa glucose-regulated protein
PDIA4_HUMAN	P13667	CK1d/e	S468	Protein disulfide-isomerase A4
KAP2_HUMAN	P13861	CK1d/g2	S80	cAMP-dependent protein kinase type II-alpha regulatory subunit
1433T_HUMAN	P27348	CK1d/e	T30	14-3-3 protein theta
1433B_HUMAN	P31946	CK1d	T32	14-3-3 protein beta/alpha
MDHC_HUMAN	P40925	CK1d/g1/g2/g3	T321/S332	Malate dehydrogenase, cytoplasmic
BAT2_HUMAN	P48634	CK1d/g1	S1092	Large proline-rich protein BAT2
TERA_HUMAN	P55072	CK1e	S37	Transitional endoplasmic reticulum ATPase
1433G_HUMAN	P61981	CK1d/e	T31	14-3-3 protein gamma
1433Z_HUMAN	P63104	CK1d/e/g1/g2/g3	S28/T30	14-3-3 protein zeta/delta
LGUL_HUMAN	Q04760	CK1d/g3	S114	Lactoylglutathione lyase
1433F_HUMAN	Q04917	CK1e/g2	T31	14-3-3 protein eta
C1QBP_HUMAN	Q07021	CK1d/e/g1/g3	S205	Complement component 1 Q subcomponent -binding protein, mitochondrial
RCN2_HUMAN	Q14257	CK1d/e/g1	T137	Reticulocalbin-2
SEPT9_HUMAN	Q9UHD8	CK1d/e	S247	Septin-9
UTP18_HUMAN	Q9Y5J1	CK1d	S121/S124/S210	U3 small nucleolar RNA-associated protein 18 homolog

### Relevance of the Substratome for Leishmania Infection

**FIGURE 2:**
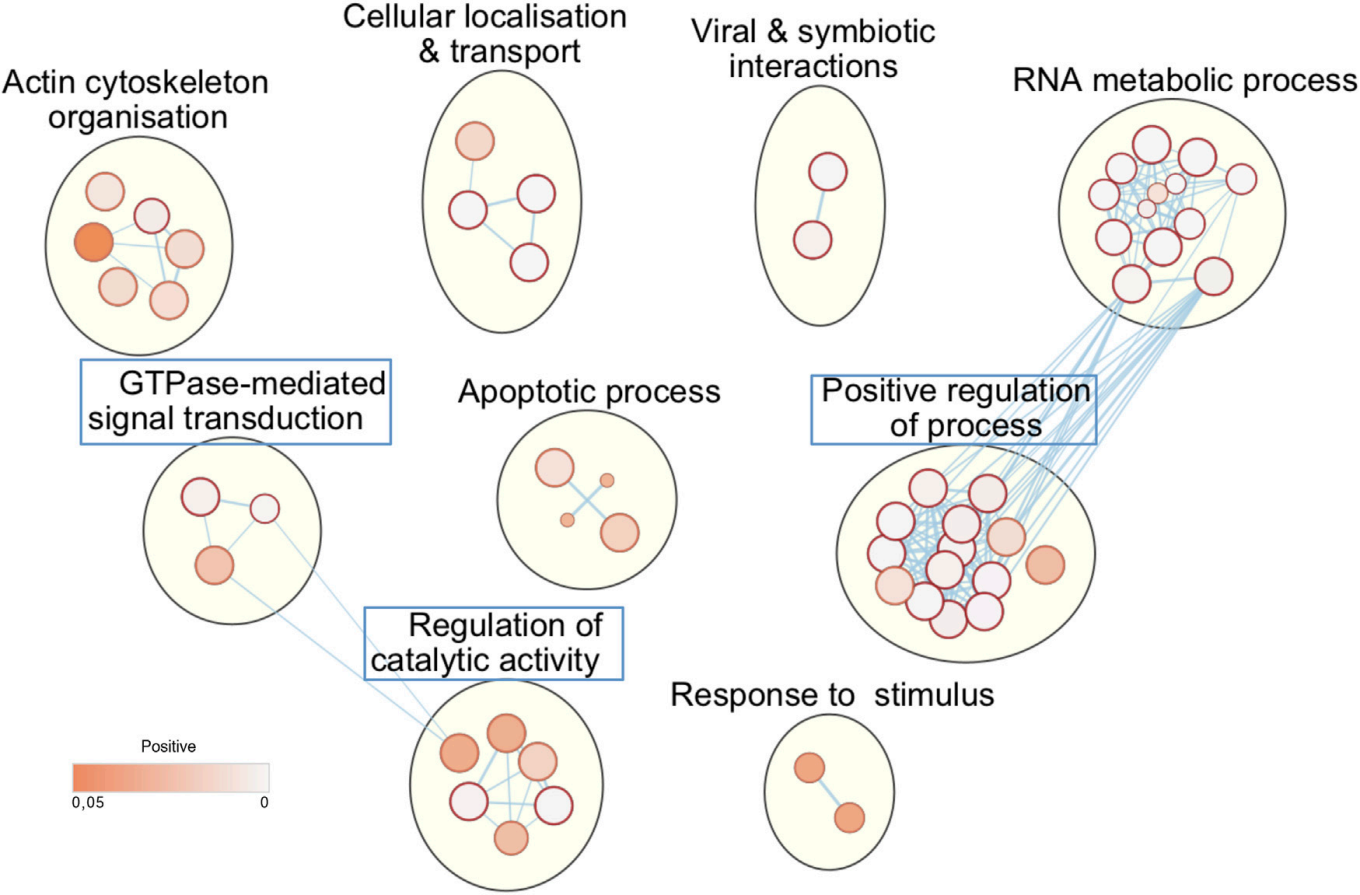
L-CK1.2 targeted biological processes. Functional enrichment analysis of the whole dataset was performed using the g:profiler web server. Results were visualized using the EnrichmentMap plugin of the Cytoscape software package, with a p-value and a Q-value above 0.05 and an edge cut-off of 0.528. Node colour represents the enrichment p-value. Node size is proportional to the total number of genes belonging to the corresponding gene-set. The edge corresponds to the Annotation shared between two nodes (blue), with edge thickness corresponding to the number of shared genes. Node clusters were identified and annotated by the AutoAnnotate plugin of cytoscape. See Supplementary Table S3 for the whole list of annotations. Blue rectangle indicates the biological processes that are specific of L-CK1.2.

Next, we asked whether the phosphorylation of these host substrates by L-CK1.2 might be relevant for *Leishmania* intracellular survival. Although, many studies described host pathways modified upon *Leishmania* infection, little is known about host proteins regulated by excreted *Leishmania* proteins and particularly excreted kinases (Isnard et al., 2012; Nandan and Reiner, 2005). Thus, we asked whether L-CK1.2 host substrates were differentially regulated during *Leishmania* infection and whether pathways enriched in our dataset were consistent with those modified during infection. Indeed, during *Leishmania* infection, 32% of L-CK1.2 substrates are differentially regulated at protein level (Smirlis et al., 2020; Singh et al., 2015; da Silva Santos et al., 2015; Negrão et al., 2019), and 30% differentially expressed at transcript level during infection (Fernandes et al., 2016; Dillon et al., 2015; Frank et al., 2015; Rabhi et al., 2013) (Table 2). These findings suggest that 60% of the proteins targeted by L-CK1.2 might be important for *Leishmania* infection. To determine the biological processes enriched in our dataset, we used Enrichment map app from cytoscape. We identified functional enrichment for nine GO terms relative to apoptosis, actin cytoskeleton organisation or RNA processing and splicing, which is consistent with *Leishmania* inhibiting host apoptosis (Moore and Matlashewski, 1994; Gupta et al., 2016), altering F-actin dynamics (de Menezes et al., 2017), or modifying the host transcriptome, respectively (Fortéa et al., 2009; Fernandes et al., 2016) (Figure 2). Moreover, several key biological processes, preferentially targeted by *Leishmania* CK1.2, are associated with host-pathogen interactions such as “viral and symbiotic interactions” or “response to stimulus” (Figure 2, Supplementary Table S3), suggesting that despite the use of non-infected macrophages as experimental system, pathways activated during infection were identified in our dataset. Altogether, this functional enrichment study suggests that the pathways targeted by L-CK1.2 *in vitro* might have some relevance *in vivo*. Further experiments will be required to determine the respective importance of all these substrates for *Leishmania* infection.

### L-CK1.2 Substratome Versus Human CK1s Substratome

**FIGURE 3:**
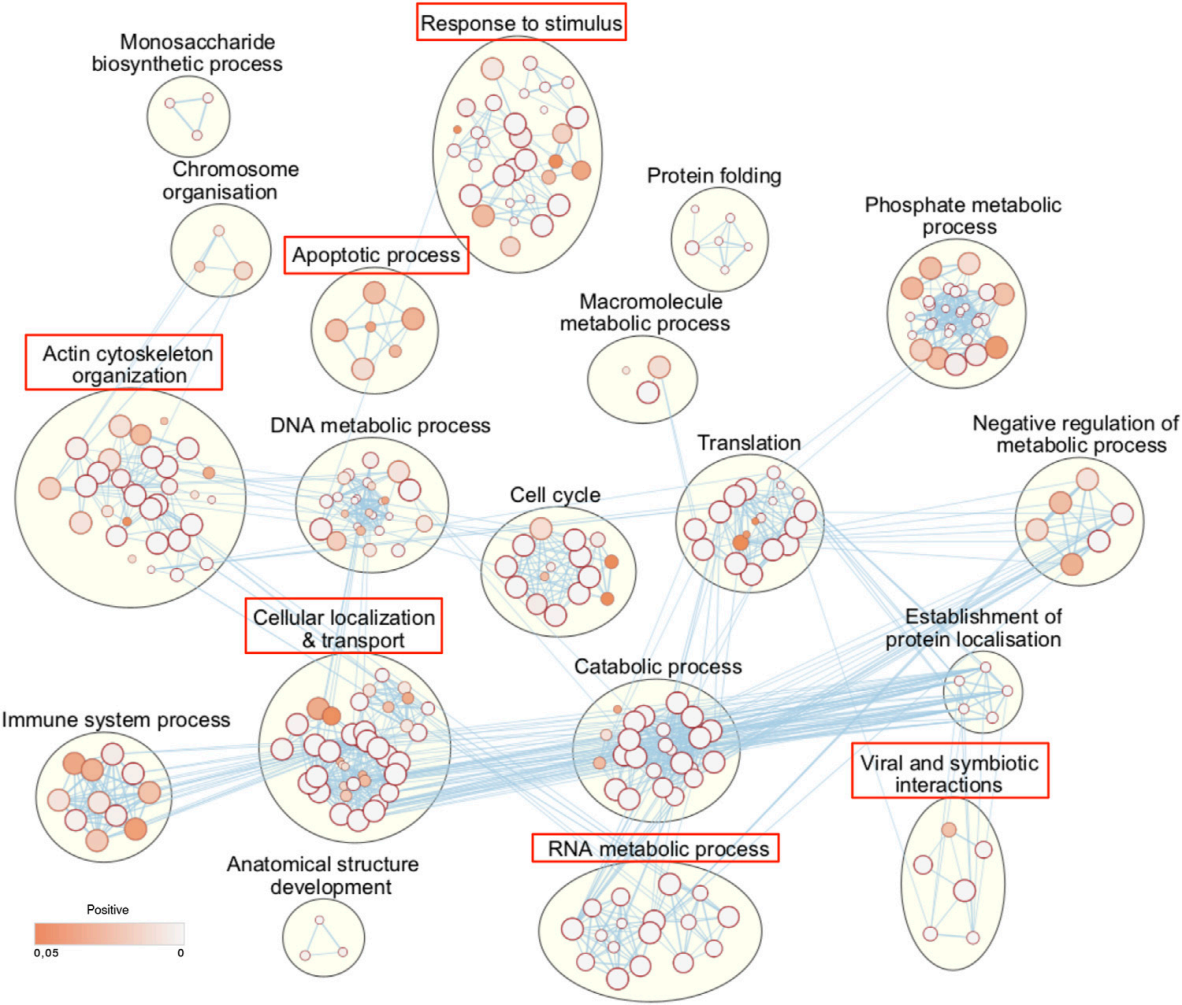
Human CK1s targeted biological processes. Functional enrichment analysis of the human CK1 substrate dataset, extracted from Sugiyama et al. (Sugiyama et al. 2019) was performed using the g:profiler web server. Results were visualized using the EnrichmentMap plugin of the Cytoscape software package, with a p-value and a Q-value above 0.05 and an edge cut-off of 0.528. Node colour represents the enrichment p-value. Node size is proportional to the total number of genes belonging to the corresponding gene-set. The edge corresponds to the Annotation shared between two nodes (blue), with edge thickness corresponding to the number of shared genes. Node clusters were identified and annotated by the AutoAnnotate plugin of cytoscape. See Supplementary Table S4 for the whole list of annotations. Red rectangle indicates the biological processes that are common with L-CK1.2 (Panel 2).

It is expected that L-CK1.2 and the host CK1s would target the same substrates, as they are closely related, but we have shown that they have structural differences in their ATP binding pocket (Durieu et al., 2016) and in their primary sequence with the presence of three additional amino acids between domain III and IV (Rachidi et al., 2014). These differences might lead to differences in substrate affinity. To examine this hypothesis and to take in account the variability of DDA of LC-MS/MS analyses, instead of comparing substrates, we compared biological process enrichments to deduce the ability of L-CK1.2 to regulate host CK1-related pathways. To this end, we took advantage of the recent substratome of human CK1s (CK1 α, δ, ε, γ1, γ2 and γ3) obtained by Sugiyama et al. Sugiyama et al. (2019) using HeLa cells, and performed a biological process enrichment map, which we compared to that of L-CK1.2 (Figure 3, Supplementary Table S4). We identified 19 groups, including “DNA metabolic process,” “cell cycle” and “catabolic process,” among which 6 are common with L-CK1.2. Noticeably, three biological processes are enriched only in L-CK1.2 dataset: “GTPase-mediated signal transduction,” “positive regulation of process” and “regulation of catalytic activity.”

### L-CK1.2 Substrates for Human Diseases

**FIGURE 4:**
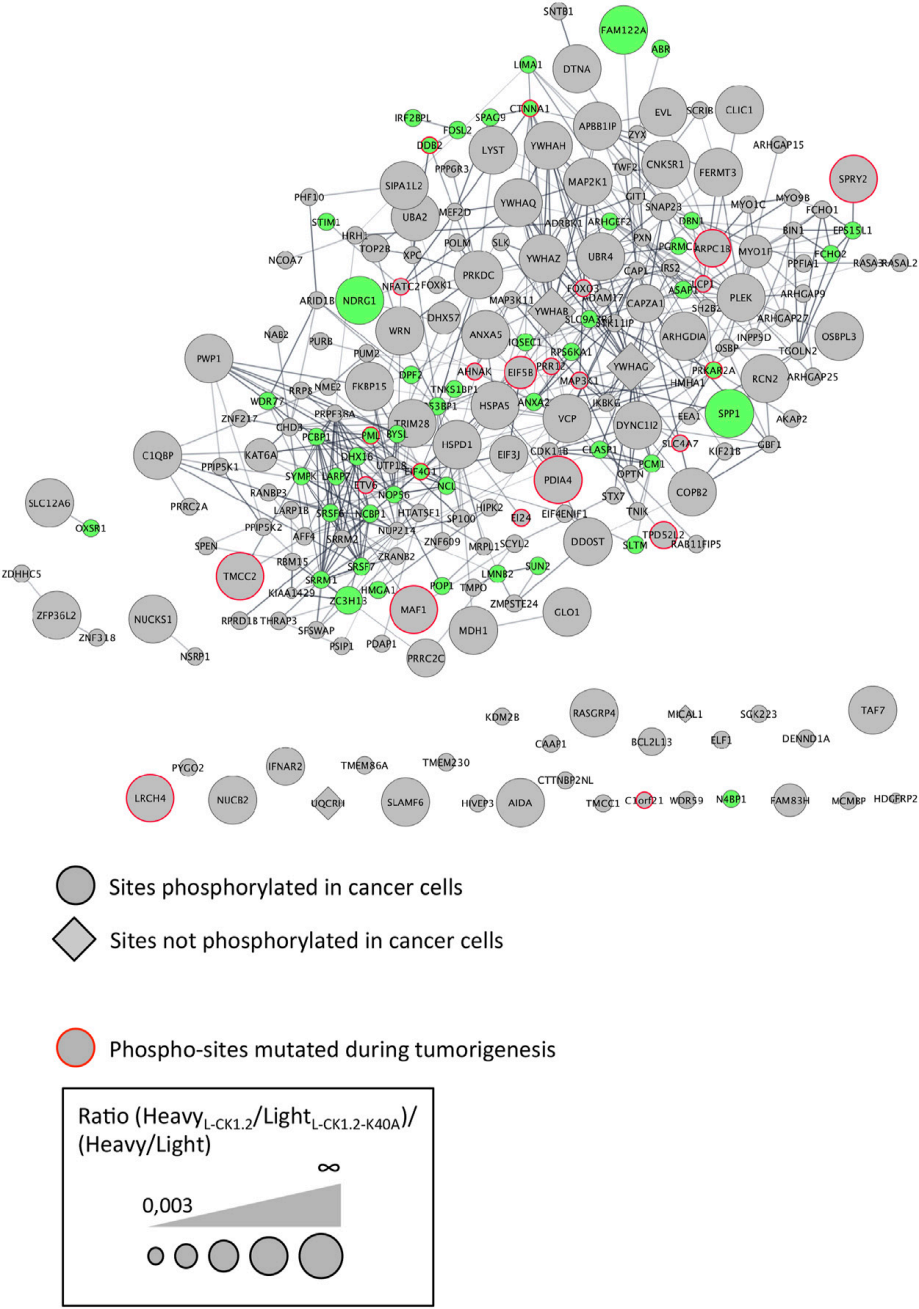
Protein-protein interaction network of L-CK1.2 substrates. The dataset was analyzed for protein-protein interactions and visualized using the STRING plugin of the Cytoscape software package. Each node represents a substrate and each edge represents a protein-protein interaction. Round shape represents sites phosphorylated in cancer cells; Diamond shape represents sites not phosphorylated in cancer cells; Red border indicates phospho-sites mutated during tumorigenesis; Green fill color indicates proteins phosphorylated during SARS-Cov2 infection. The labeling indicates the UniProt human name. The size of the node represents the ratio (Heavy_L-CK1.2_/Light_L-CK1.2-K40A_)/(Heavy/Light mock).

CK1 family members have been implicated in the physiopathology of several human diseases such as cancer or infectious diseases (Knippschild et al., 2014; Xu et al., 2019). Indeed, defect in CK1 regulation is associated with cancer, while survival of intracellular pathogens relies on the manipulation of host CK1-pathways (Jayaswal et al., 2010; Cegielska and Virshup, 1993; Zhang et al., 2017). One intriguing possibility would be that the modification of similar CK1 pathways might contribute to the pathology of these different diseases. To support this hypothesis, we investigated whether any of L-CK1.2 substrates might be important for other human diseases, focusing on cancer and viral infection. First, we showed using PhosphoSitePlus that 1) 76% of the L-CK1.2 substrates are considered as prognosis markers for various cancer types (Supplementary Table S1), 2) 89% of L-CK1.2 phosphosites are phosphorylated in cancer cells (Table 1; Supplementary Table S1 and Figure 4, round shape), and 3) 10% of L-CK1.2 phosphosites are mutated during tumorigenesis (Table 1; Supplementary Table S1 and Figure 4, red border). These data are consistent with the fact that human CK1s are overexpressed in cancer cells (Xu et al., 2019). Second, we showed that 51% of L-CK1.2 substrates are phosphorylated during SARS-CoV2 infection (Figure 4, green, Table 2 and Supplementary Table S1), and 20% on the same sites as those phosphorylated by L-CK1.2 (Table 1 and Supplementary Table S1) (Bouhaddou et al., 2020). Only two biological processes, “RNA splicing” and “cellular localisation and transport,” are commonly enriched (Supplementary Figure S3 and Supplementary Tables S5, S6). Interestingly, L-CK1.2 substratome contains eight proteins that interact with SARS-CoV2 proteins, suggesting similar regulations (Gordon et al., 2020). Altogether, our data point to similar pathways being altered in different human diseases.

## Discussion

Increasing the knowledge on released parasite proteins is crucial to better understand host-pathogen interactions. Even more when studying host cell signalling pathways exploited by pathogens during infection through the release of their kinases, hence the importance of finding their host substrates in an unbiased manner. Here, we developed a method, applicable to other kinases, combining SILAC-based quantitative mass spectrometry, pulsed heating and IVKA that allows direct detection of phosphorylation and does not require the modification of the kinase (Shah et al., 1997). We applied this method to L-CK1.2, as, despite its essentialness for intracellular parasite survival, little is known on the host functions of this signalling kinase released by *Leishmania*, except its phosphorylation of host IFNAR1, the receptor to interferon α/β (Liu et al., 2009). We identified 257 phospho-sites corresponding to 225 substrates. Although our approach has limitations inherent to the variability of the DDA of mass spectrometry analyses or to the IVKA, we validated them as *bona fide* CK1 substrates, suggesting that the above limitations did not compromised our ability to identify true L-CK1.2 substrates. Regarding the physiological relevance of those substrates, we cannot exclude that the pulsed heating might have altered protein structure and revealed sites that would normally not be accessible. However, we do not favour this hypothesis for two reasons. First, most of the phosphosites in our dataset are phosphorylated *in vivo* (PhosphoSitePlus), suggesting that they are accessible even integrated into protein complexes*.* Second, we validated experimentally some of these substrates even when correctly folded. These results will need experimental confirmation in a cellular model, nonetheless they provide pathways to prioritise for further characterisation. We showed that only few substrates are common to the two datasets, + and - phosphatase treatment, which can be explained as follows. 1) Substrates carrying the following consensus site, [S/T]pX_2-3_ [**pS**/**pT**], might have been lost from the “+ phosphatase” dataset, as it requires priming. 2) Phospho-peptides were lost as a consequence of the variability in TiO_2_ purification, of DDA LC-MS/MS analysis. 3) Dephosphorylation might have made accessible residues for which L-CK1.2 has more affinity and thus might phosphorylate preferentially when available, explaining why some residues targeted efficiently in absence of phosphatase treatment were no longer identified after the treatment. These variabilities led to the lost of a substantial number of common substrates, as they were above threshold in only one of the three replicates, which is not sufficient to be considered as a substrate.

From our large dataset, we showed that L-CK1.2 phosphorylates the known canonical and non-canonical CK1 recognition motifs, which is consistent with previous observations (Rachidi et al., 2014). The only differences are first linked to the motif identified by Kawakami *et al.* K/R(X)K/R (XX)**pS**/**pT** (Kawakami et al., 2008), as our results suggest a shorter motif, K/R (XX)**pS**/**pT**. Our analysis is based on a bigger number of substrates, which might explain the difference with Kawakami et al. (2008). The second difference is the presence of a proline residue adjacent to the phosphorylated S/T ([**pS**/**pT**][P]). Sugiyama *et al.* identified 507 substrates for human CK1α, δ, ε, γ1, γ2, γ3, and found similar CK1 phosphorylation motifs to those we identified with the exception of the proline in +1 (Sugiyama et al., 2019). L-CK1.2 might have more affinity for consensus sites containing a proline adjacent to the S/T, which might be a way to restrict the host CK1 substrates targeted. Its requirement and its importance for L-CK1.2 substrate affinity remain to be confirmed experimentally. Furthermore, we identified a potential new CK1 consensus site, [G]X_2-3_ [**pS**/**pT**], which was validated experimentally with L-CK1.2 and CK1δ (SNAP23, Figures 1C,D). Further analyses, including mutagenesis, will be required to confirm this new consensus, as we cannot exclude the possibility that SNAP23 was phosphorylated on another site. L-CK1.2, not only targets similar consensus sites as mammalian CK1s but also similar biological processes (BP), as we showed that five human CK1 BP were also enriched in L-CK1.2 dataset. Noticeably, L-CK1.2 seems to target fewer pathways than human CK1s. Although we cannot rule out that it might be the consequence of the variability inherent to proteomic analyses, L-CK1.2 and human CK1s might have differences in substrate affinity despite high level of identity. Indeed, structural differences (Rachidi et al., 2014; Durieu et al., 2016) and a potentially more restricted consensus site (presence of a proline, this work) might explain this specificity. Further analyses will be required to distinguish between these two possibilities.

Numerous publications described host pathways modified during *Leishmania* infection but only few established a link between these pathways and parasite effectors. Our study by determining the host substrates of *Leishmania* CK1.2 highlight, for the first time, the pathways it may regulate in the host cell, providing a potential new link between a parasite effector and the host pathways it modifies. These pathways are consistent with those modified during *Leishmania* infection. For instance, several studies have shown that *Leishmania* infection inhibits macrophage apoptosis, which might contribute to the spread of the infection and parasite transmission (Moore and Matlashewski, 1994; Gupta et al., 2016). Indeed, we identified 41 L-CK1.2 host substrates involved in apoptosis, such as BCL2L13, which regulates the role of mitochondria-mediated cell death pathway and can be pro- or anti-apoptotic (Meng et al., 2021), or FOXO3, which is a transcription factor inducing transcription of genes involved in apoptosis. There are other examples with host pathways such as “actin cytoskeleton organisation’ or RNA metabolic process” that are both targeted by L-CK1.2 and modified during *Leishmania* infection (Fernandes et al., 2016; de Menezes et al., 2017; Smirlis et al., 2020; Bichiou et al., 2021). Extensive work remains to be done to ascertain the link between L-CK1.2 and host pathways modified during *Leishmania* infection. Nevertheless, the biological processes enriched among L-CK1.2 host substrates provide a starting point to prioritize pathways that should be characterised to reveal L-CK1.2 functions in the host cell. Furthermore, our dataset provides a list of potential host targets for host-targeted therapy against leishmaniasis (Lamotte et al., 2017).

Finally, although seemingly different, Visceral Leishmaniasis (VL), COVID-19 and cancer have manifestations that are to some extent common. For example, the uncontrolled overproduction of cytokines, namely the cytokine storm, is a common feature of terminal VL (Santos-Oliveira et al., 2011), SARS-CoV-2 infection and cancer (Turnquist et al., 2020). At the cellular level, similar pathways are regulated in infected or cancer cells. For instance, apoptosis is inhibited in *Leishmania* infected macrophages as well as in cancer cells (Knippschild et al., 2014; Gupta et al., 2016). The immune response is attenuated, preventing infected or cancer cells from being destroyed by the immune system (Knippschild et al., 2014; Figueiredo et al., 2016). CK1 is involved in the regulation of these two pathways, suggesting that it might be important for disease development (Knippschild et al., 2014; Xu et al., 2019; Rachidi et al., 2021). Indeed, human CK1 has cancer-associated functions linked to its involvement in the Wnt (Wingless/Int-1), Hh (Hedgehog), and Hippo signalling pathways (Knippschild et al., 2014; Xu et al., 2019), as well as an involvement in viral, bacterial and parasitic infections (Cegielska and Virshup, 1993; Jayaswal et al., 2010; Durieu et al., 2016; Zhang et al., 2017). Therefore CK1 substrates, including those identified in this work, might be similarly altered in different diseases. For instance, inactivation of FOXO3 is associated with the initiation and progression of cancer (Liu et al., 2018) and has a role in infectious diseases through regulation of IL-10 (Kane and Mosser, 2001; Bouzeyen et al., 2019). Understanding these molecular connections between infectious diseases and cancer may help health care providers discover new therapies for combatting these diseases.

## Data Availability

The datasets presented in this study can be found in online repositories. The names of the repository/repositories and accession number(s) can be found below: https://www.ebi.ac.uk/pride/archive/, PXD026220.

## Supplementary Material

Supplementary Figure S1 Increase of accessible residues for *de novo* phosphorylation following phosphatase treatment. Left panel Autoradiogram representing IVKA using THP-1 cell extracts treated (lane 3 and 4) or not (lane 1 and 2) with Antarctic phosphatase, and active L-CK1.2 (lane 1 and 3) or inactive L-CK1.2-K40A (lane 2 and 4). Right Panel Same gel stained with coomassie blue.

Supplementary Figure S2 Comparison of the different experimental conditions. Venn diagrams showing the numbers and overlaps of phospho-peptides **(A,C)** or proteins **(B,D)** in the following four experiments: Heavy_L-CK1.2_/Light_L-CK1.2-K40A_ and Heavy/Light mock, treated **(A,B)** or not with Antarctic phosphatase **(C,D)**.

Supplementary Figure S3 Common pathways targeted by L-CK1.2 and phosphorylated during SARS-CoV2 infection. Functional enrichment analysis of the proteins phosphorylated by L-CK1.2 and also during SARS-CoV2 infection was performed using the g:profiler web server. Results were visualized using the EnrichmentMap plugin of the Cytoscape software package, with a p-value and a Q-value above 0.03 and an edge cut-off of 0.375. Node colour represents the enrichment p-value. Node size is proportional to the total number of genes belonging to the corresponding gene-set. The edge corresponds to the Annotation shared between two nodes (blue), with edge thickness corresponding to the number of shared genes. Node clusters were identified and annotated by the AutoAnnotate plugin of cytoscape. See Table 2 (RNA splicing and processing) and 3 (cellular localisation & transport) for the list of annotations.
