# Supplementary material for: Identification of the Host Substratome of Leishmania-Secreted Casein Kinase 1 Using a SILAC-Based Quantitative Mass Spectrometry Assay
Source: Front Cell Dev Biol. 2022 Jan 3;9:800098. doi: 10.3389/fcell.2021.800098 (PMC8762337; doi:10.3389/fcell.2021.800098)
Supplement: Supplementary file 1 [file DataSheet1.PDF]

Figure S1

A

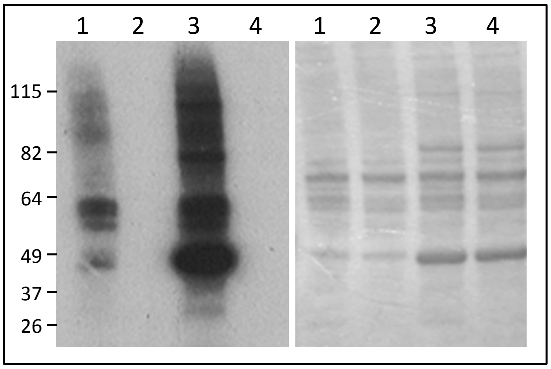

Figure S2

A

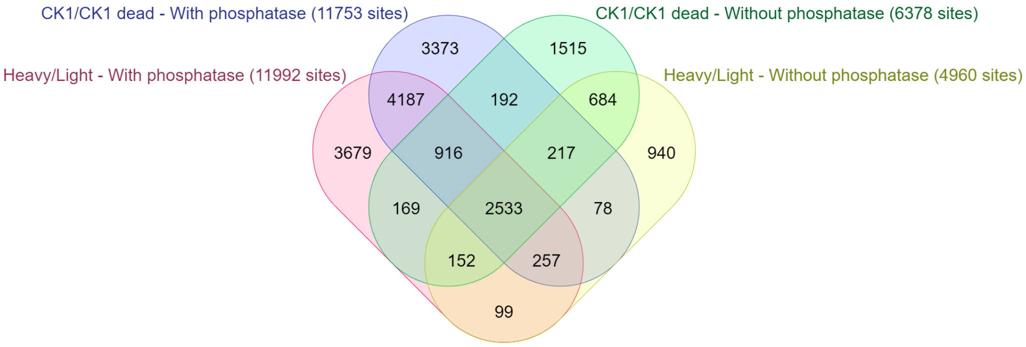

B

	CK1/CK1 dead - Wit h phosphatase (11753)	CK1/CK1 dead - Wit hout phosphatase (6378)	Heavy/Light - Withou t phosphatase (4960)
Heavy/Light - With phosphatase (11992)	7893	3770	3041
CK1/CK1 dead - With phosphatase (11753)		3858	3085
CK1/CK1 dead - Without phosphatase (6378)			3586

C

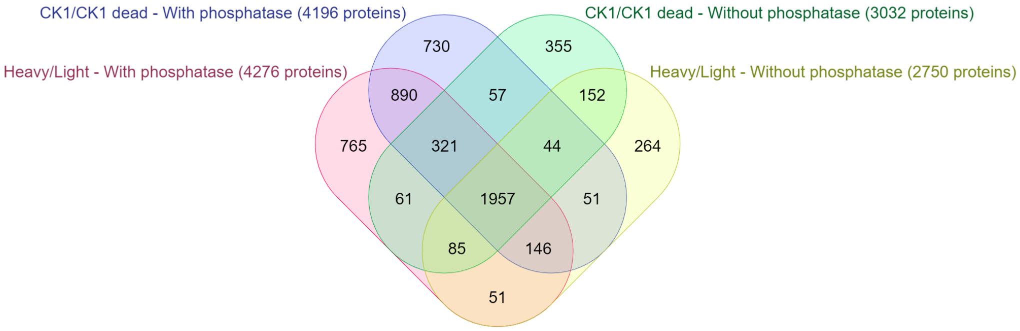

D

	CK1/CK1 dead - Wit h phosphatase (4196)	CK1/CK1 dead - Wit hout phosphatase (3032)	Heavy/Light - Withou t phosphatase (2750)
Heavy/Light - With phosphatase (4276)	3314	2424	2239
CK1/CK1 dead - With phosphatase (4196)		2379	2198
CK1/CK1 dead - Without phosphatase (3032)			2238

Figure S3

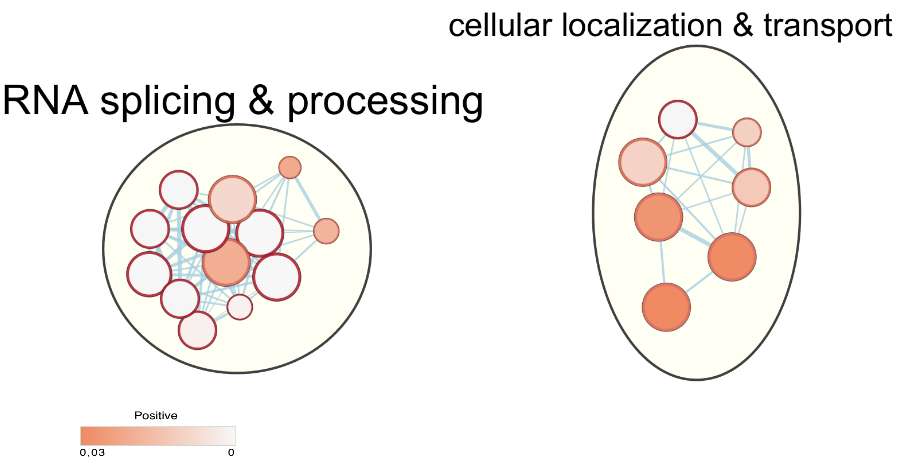
cellular localization & transport
RNA splicing & processing
Positive
0,03
0

Table S2: List of L-CK1.2 host substrates also phosphorylated by huCK1s

Substrate/Uniprot ID	huCK1 Kinase	uniprot	Protein description
CLIC1_HUMAN	CK1d/e	O00299	Chloride intracellular channel protein 1
ARC1B_HUMAN	CK1d	O15143	Actin-related protein 2/3 complex subunit 1B
TPD54_HUMAN	CK1d	O43399	Tumor protein D54
HTSF1_HUMAN	CK1a/d/e/g3	O43719	HIV Tat-specific factor 1
DHX16_HUMAN	CK1d/e/g1/g3	O60231	Putative pre-mRNA-splicing factor ATP-dependent RNA helicase DHX16
IF2P_HUMAN	CK1a/d/e/g1/g3	O60841	Eukaryotic translation initiation factor 5B
EIF3J_HUMAN	CK1d	O75822	Eukaryotic translation initiation factor 3 subunit J
ZRAB2_HUMAN	CK1a/d/e/g1/g2/g3	O95218	Zinc finger Ran-binding domain-containing protein 2
OXSR1_HUMAN	CK1d	O95747	Serine/threonine-protein kinase OSR1
ANXA2_HUMAN	CK1a/d/e/g1/g2/g3	P07355	Annexin A2
ANXA5_HUMAN	CK1d/e	P08758	Annexin A5
CH60_HUMAN	CK1d/e	P10809	60 kDa heat shock protein, mitochondrial
GRP78_HUMAN	CK1a/d/e/g1/g2/g3	P11021	78 kDa glucose-regulated protein
PDIA4_HUMAN	CK1d/e/g2/g3	P13667	Protein disulfide-isomerase A4
KAP2_HUMAN	CK1a/d/g2	P13861	cAMP-dependent protein kinase type II-alpha regulatory subunit
NUCL_HUMAN	CK1a/d/e/g1/g2/g3	P19338	Nucleolin
OSBP1_HUMAN	CK1a/g2	P22059	Oxysterol-binding protein 1
1433T_HUMAN	CK1a/d/e	P27348	14-3-3 protein theta
1433B_HUMAN	CK1a/d	P31946	14-3-3 protein beta/alpha
MDHC_HUMAN	CK1d/e/g1/g2/g3	P40925	Malate dehydrogenase, cytoplasmic
BAT2_HUMAN	CK1d/g1/g2	P48634	Large proline-rich protein BAT2
TERA_HUMAN	CK1d/e/g2	P55072	Transitional endoplasmic reticulum ATPase
1433G_HUMAN	CK1a/d/e	P61981	14-3-3 protein gamma
1433Z_HUMAN	CK1a/d/e/g1/g2/g3	P63104	14-3-3 protein zeta/delta
IF4G1_HUMAN	CK1d	Q04637	Eukaryotic translation initiation factor 4 gamma 1
LGUL_HUMAN	CK1d/g3	Q04760	Lactoylglutathione lyase
1433F_HUMAN	CK1a/d/e/g2	Q04917	14-3-3 protein eta
C1QBP_HUMAN	CK1d/e/g1/g3	Q07021	Complement component 1 Q subcomponent-binding protein, mitochondrial
TP53B_HUMAN	CK1g2/g3	Q12888	Tumor suppressor p53-binding protein 1
TIF1B_HUMAN	CK1a/d/e/g2/g3	Q13263	Transcription intermediary factor 1-beta
PWP1_HUMAN	CK1d	Q13610	Periodic tryptophan protein 1 homolog
RCN2_HUMAN	CK1d/e/g1	Q14257	Reticulocalbin-2
UBR4_HUMAN	CK1g2	Q5T4S7	E3 ubiquitin-protein ligase UBR4
CLAP1_HUMAN	CK1d/g2	Q7Z460	CLIP-associating protein 1
HDGR2_HUMAN	CK1a	Q7Z4V5	Hepatoma-derived growth factor-related protein 2
REQU_HUMAN	CK1g2/g3	Q92785	Zinc finger protein ubi-d4
ARHG2_HUMAN	CK1g3	Q92974	Rho guanine nucleotide exchange factor 2
MINT_HUMAN	CK1g3	Q96T58	Msx2-interacting protein
TB182_HUMAN	CK1a/d/e/g2	Q9C0C2	182 kDa tankyrase-1-binding protein
NUCKS_HUMAN	CK1a/d/e/g2/g3	Q9H1E3	Nuclear ubiquitous casein and cyclin-dependent kinases substrate
SLK_HUMAN	CK1e1g3	Q9H2G2	STE20-like serine/threonine-protein kinase
RANB3_HUMAN	CK1d	Q9H6Z4	Ran-binding protein 3
SEPT9_HUMAN	CK1d	Q9UHD8	Septin-9
GIT1_HUMAN	CK1d	Q9Y2X7	ARF GTPase-activating protein GIT1
BA2L2_HUMAN	CK1a/d/g3	Q9Y520	Protein BAT2-like 2
UTP18_HUMAN	CK1d	Q9Y5J1	U3 small nucleolar RNA-associated protein 18 homolog

Table S3: List of annotations of L-CK1.2 host substrates enrichment map (Figure 3)

Gene set name	Gene set description	fdr_qvalue
Actin cytoskeleton organisation		
GO:0030036	actin cytoskeleton organization	0.010148642
GO:0007015	actin filament organization	0.003986568
GO:0030029	actin filament-based process	0.009912871
GO:0044085	cellular component biogenesis	0.011134548
GO:0006996	organelle organization	0.045068974
GO:0051128	regulation of cellular component organization	0.006282436
Viral & symbiotic interactions		
GO:0044403	biological process involved in symbiotic interaction	0.002458985
GO:0016032	viral process	6.70E-04
Response to stimulus		
GO:0071214	cellular response to abiotic stimulus	0.033084627
GO:0104004	cellular response to environmental stimulus	0.033084627
GTPase-mediated signal transduction		
GO:0035556	intracellular signal transduction	0.020143175
GO:0051056	regulation of small GTPase mediated signal transduction	0.001020463
GO:0007264	small GTPase mediated signal transduction	0.002340487
Cellular localisation and transport		
GO:0051641	cellular localization	1.52E-06
GO:0051649	establishment of localization in cell	1.11E-06
GO:0046907	intracellular transport	9.95E-05
GO:1903827	regulation of cellular protein localization	0.012139707
Apoptosis process		
GO:0006915	apoptotic process	0.008010955
GO:0008219	cell death	0.015430219
GO:1900740	positive regulation of protein insertion into mitochondrial membrane involved in apoptotic signaling pathway	0.02742253
GO:1900739	regulation of protein insertion into mitochondrial membrane involved in apoptotic signaling pathway	0.02742253
Regulation of catalytic activity		
GO:0043085	positive regulation of catalytic activity	0.029701682
GO:0043547	positive regulation of GTPase activity	0.022879605
GO:0044093	positive regulation of molecular function	0.001917564
GO:0050790	regulation of catalytic activity	0.014427716
GO:0065009	regulation of molecular function	7.54E-04
GO:0048583	regulation of response to stimulus	0.030720627
Positive regulation of process		
GO:0031324	negative regulation of cellular metabolic process	0.02340261
GO:0009891	positive regulation of biosynthetic process	0.004134998
GO:0031328	positive regulation of cellular biosynthetic process	0.002433485
GO:0031325	positive regulation of cellular metabolic process	2.83E-04
GO:0010557	positive regulation of macromolecule biosynthetic process	0.002615297
GO:0010604	positive regulation of macromolecule metabolic process	0.003190292
GO:0009893	positive regulation of metabolic process	0.008312924
GO:0051173	positive regulation of nitrogen compound metabolic process	0.003447023
GO:1903508	positive regulation of nucleic acid-templated transcription	0.001055616
GO:0045935	positive regulation of nucleobase-containing compound metabolic process	7.26E-04
GO:1902680	positive regulation of RNA biosynthetic process	0.001073438
GO:0051254	positive regulation of RNA metabolic process	2.91E-04
GO:0045893	positive regulation of transcription, DNA-templated	0.001055616
GO:0019219	regulation of nucleobase-containing compound metabolic process	0.010585512
RNA metabolic process		
GO:1903311	regulation of mRNA metabolic process	2.49E-05
GO:0050684	regulation of mRNA processing	1.27E-04
GO:0048024	regulation of mRNA splicing, via spliceosome	0.003665786
GO:0051252	regulation of RNA metabolic process	0.002945695
GO:0043484	regulation of RNA splicing	0.008004015
GO:0016070	RNA metabolic process	9.47E-04
GO:0006396	RNA processing	6.30E-07
GO:0008380	RNA splicing	2.89E-06
GO:0000375	RNA splicing, via transesterification reactions	3.62E-05
GO:0000377	RNA splicing, via transesterification reactions with bulged adenosine as nucleophile	3.15E-05
GO:0016071	mRNA metabolic process	2.61E-06
GO:0006397	mRNA processing	2.73E-06
GO:0000398	mRNA splicing, via spliceosome	3.15E-05

Table S4: List of annotations of human CK1s substrates enrichment map (Figure 4)

Gene set name	Gene set description	fdr_qvalue
Monosaccharide biosynthetic process		
GO:0019319	hexose biosynthetic process	0.002838062
GO:0006094	gluconeogenesis	0.001932891
GO:0046364	monosaccharide biosynthetic process	9.51E-04
Macromolecule metabolic process		
GO:0010628	positive regulation of gene expression	1.23E-04
GO:0010604	positive regulation of macromolecule metabolic process	0.011072471
GO:0090131	mesenchyme migration	0.006200006
Chromosome organisation		
GO:0071103	DNA conformation change	0.011659816
GO:0032508	DNA duplex unwinding	0.017332116
GO:0032392	DNA geometric change	0.005427524
Anatomical structure development		
GO:0048857	neural nucleus development	0.00320186
GO:0021762	substantia nigra development	8.21E-05
GO:0030901	midbrain development	0.002838062
Establishment of protein localisation		
GO:0072599	establishment of protein localization to endoplasmic reticulum	6.99E-15
GO:0045047	protein targeting to ER	2.60E-15
GO:0006614	SRP-dependent cotranslational protein targeting to membrane	8.56E-15
GO:0070972	protein localization to endoplasmic reticulum	3.41E-14
GO:0006613	cotranslational protein targeting to membrane	4.19E-14
Viral and symbiotic interactions		
GO:0019080	viral gene expression	1.88E-17
GO:0019058	viral life cycle	4.13E-05
GO:0016032	viral process	2.82E-28
GO:0051701	biological process involved in interaction with host	0.023058426
GO:0044403	biological process involved in symbiotic interaction	2.17E-04
GO:0019083	viral transcription	4.00E-14
Protein folding		
GO:0051084	'de novo' posttranslational protein folding	4.44E-10
GO:0006458	'de novo' protein folding	1.14E-10
GO:0042026	protein refolding	1.14E-08
GO:0051085	chaperone cofactor-dependent protein refolding	1.02E-09
GO:0006457	protein folding	4.94E-17
GO:0061077	chaperone-mediated protein folding	1.14E-09
Negative regulation of metabolic process		
GO:0009890	negative regulation of biosynthetic process	0.00765959
GO:0031327	negative regulation of cellular biosynthetic process	0.025163549
GO:2000113	negative regulation of cellular macromolecule biosynthetic process	0.030520534
GO:0010558	negative regulation of macromolecule biosynthetic process	0.011072052
GO:0010605	negative regulation of macromolecule metabolic process	6.50E-07
GO:0009892	negative regulation of metabolic process	1.84E-06
Apoptotic process		
GO:0042981	regulation of apoptotic process	0.03012088
GO:0043066	negative regulation of apoptotic process	0.011220554
GO:0043069	negative regulation of programmed cell death	0.022380214
GO:2001242	regulation of intrinsic apoptotic signaling pathway	0.027571126
GO:2001243	negative regulation of intrinsic apoptotic signaling pathway	0.036590777
GO:0006915	apoptotic process	0.024040044
Immune system process		
GO:0002275	myeloid cell activation involved in immune response	0.038611063
GO:0002444	myeloid leukocyte mediated immunity	0.019195787
GO:0016192	vesicle-mediated transport	0.002865296
GO:0002263	cell activation involved in immune response	0.034545388
GO:0042119	neutrophil activation	0.005264233
GO:0002283	neutrophil activation involved in immune response	0.002838143
GO:0043312	neutrophil degranulation	0.002260413
GO:0002446	neutrophil mediated immunity	0.004827836
GO:0002366	leukocyte activation involved in immune response	0.030405357
GO:0043299	leukocyte degranulation	0.022807547
GO:0036230	granulocyte activation	0.00709532

Table S4: List of annotations of human CK1s substrates enrichment map (Figure 4)

Gene set name	Gene set description	fdr_qvalue
Cell cycle		
GO:0036388	pre-replicative complex assembly	0.00320186
GO:1902850	microtubule cytoskeleton organization involved in mitosis	0.047898554
GO:0007051	spindle organization	0.004071501
GO:0007049	cell cycle	1.13E-06
GO:0044770	cell cycle phase transition	5.92E-05
GO:0022402	cell cycle process	1.57E-06
GO:0010458	exit from mitosis	0.025424707
GO:0051726	regulation of cell cycle	0.001179617
GO:1901987	regulation of cell cycle phase transition	0.010711129
GO:0010564	regulation of cell cycle process	1.03E-04
GO:1901990	regulation of mitotic cell cycle phase transition	0.004705085
GO:0000278	mitotic cell cycle	2.48E-08
GO:0044772	mitotic cell cycle phase transition	5.86E-05
GO:1903047	mitotic cell cycle process	1.65E-06
GO:0007052	mitotic spindle organization	0.047924541
RNA metabolic process		
GO:0016071	mRNA metabolic process	3.34E-34
GO:0006397	mRNA processing	2.26E-08
GO:0000398	mRNA splicing, via spliceosome	1.17E-07
GO:0016070	RNA metabolic process	6.15E-08
GO:0006396	RNA processing	2.80E-19
GO:0000375	RNA splicing, via transesterification reactions	3.47E-08
GO:0000377	RNA splicing, via transesterification reactions with bulged adenosine as nucleophile	1.17E-07
GO:0008380	RNA splicing	1.29E-10
GO:0016072	rRNA metabolic process	1.78E-07
GO:0006364	rRNA processing	7.03E-08
GO:0034660	ncRNA metabolic process	1.26E-10
GO:0034470	ncRNA processing	2.13E-09
GO:0050684	regulation of mRNA processing	7.51E-07
GO:0048024	regulation of mRNA splicing, via spliceosome	6.25E-05
GO:0042254	ribosome biogenesis	3.30E-09
GO:0022613	ribonucleoprotein complex biogenesis	3.51E-16
GO:0043484	regulation of RNA splicing	1.83E-06
Translation		
GO:0006446	regulation of translational initiation	0.004459547
GO:1901566	organonitrogen compound biosynthetic process	5.61E-13
GO:0032268	regulation of cellular protein metabolic process	9.74E-06
GO:0002181	cytoplasmic translation	6.26E-28
GO:0006413	translational initiation	3.57E-25
GO:1903608	protein localization to cytoplasmic stress granule	0.041578536
GO:0017148	negative regulation of translation	0.048508525
GO:0006417	regulation of translation	3.53E-09
GO:0034248	regulation of cellular amide metabolic process	9.91E-08
GO:0006412	translation	3.68E-24
GO:0043603	cellular amide metabolic process	1.25E-15
GO:0043604	amide biosynthetic process	1.05E-19
GO:0010608	posttranscriptional regulation of gene expression	7.99E-11
GO:0002183	cytoplasmic translational initiation	4.32E-05
GO:0097010	eukaryotic translation initiation factor 4F complex assembly	0.047627289
GO:0043043	peptide biosynthetic process	4.72E-23
GO:0051246	regulation of protein metabolic process	5.21E-05
GO:0006518	peptide metabolic process	7.39E-21
GO:0019081	viral translation	2.11E-06

Table S4: List of annotations of human CK1s substrates enrichment map (Figure 4)

Gene set name	Gene set description	fdr_qvalue
Catabolic process		
GO:0006402	mRNA catabolic process	3.26E-26
GO:0061013	regulation of mRNA catabolic process	9.02E-10
GO:0000184	nuclear-transcribed mRNA catabolic process, nonsense-mediated decay	3.37E-16
GO:0044265	cellular macromolecule catabolic process	1.49E-16
GO:0044270	cellular nitrogen compound catabolic process	3.85E-22
GO:2000058	regulation of ubiquitin-dependent protein catabolic process	0.025547171
GO:0009894	regulation of catabolic process	1.10E-05
GO:0034655	nucleobase-containing compound catabolic process	1.32E-23
GO:0035973	aggrephagy	0.035441938
GO:1901575	organic substance catabolic process	8.36E-14
GO:0043488	regulation of mRNA stability	2.12E-09
GO:0044248	cellular catabolic process	1.68E-10
GO:0010629	negative regulation of gene expression	9.02E-13
GO:0046700	heterocycle catabolic process	2.14E-22
GO:0031329	regulation of cellular catabolic process	7.60E-07
GO:0006401	RNA catabolic process	3.52E-26
GO:0019439	aromatic compound catabolic process	1.34E-21
GO:1903311	regulation of mRNA metabolic process	1.72E-16
GO:1901361	organic cyclic compound catabolic process	3.52E-21
GO:0000956	nuclear-transcribed mRNA catabolic process	1.18E-13
GO:0009056	catabolic process	1.26E-11
GO:0009057	macromolecule catabolic process	4.58E-13
GO:0043487	regulation of RNA stability	1.39E-09
GO:0032434	regulation of proteasomal ubiquitin-dependent protein catabolic process	0.00844825
DNA metabolic process		
GO:0071897	DNA biosynthetic process	2.78E-04
GO:0006278	RNA-dependent DNA biosynthetic process	2.97E-04
GO:0060249	anatomical structure homeostasis	0.007276978
GO:0050821	protein stabilization	7.53E-04
GO:0006259	DNA metabolic process	0.001915153
GO:2000573	positive regulation of DNA biosynthetic process	0.027135716
GO:0051054	positive regulation of DNA metabolic process	0.005137974
GO:1904851	positive regulation of establishment of protein localization to telomere	0.007581187
GO:1904871	positive regulation of protein localization to Cajal body	3.67E-04
GO:1904816	positive regulation of protein localization to chromosome, telomeric region	0.036218918
GO:1904358	positive regulation of telomere maintenance via telomere lengthening	0.012105713
GO:1904867	protein localization to Cajal body	7.18E-04
GO:1903405	protein localization to nuclear body	7.18E-04
GO:1990173	protein localization to nucleoplasm	0.002228041
GO:0034504	protein localization to nucleus	0.003448312
GO:0065008	regulation of biological quality	4.57E-05
GO:0051052	regulation of DNA metabolic process	0.016828729
GO:0070202	regulation of establishment of protein localization to chromosome	0.022789563
GO:0070203	regulation of establishment of protein localization to telomere	0.013592919
GO:1904869	regulation of protein localization to Cajal body	3.67E-04
GO:0031647	regulation of protein stability	0.005531697
GO:0000723	telomere maintenance	0.00168719
GO:0007004	telomere maintenance via telomerase	2.15E-04
GO:0010833	telomere maintenance via telomere lengthening	1.82E-04
GO:0032200	telomere organization	0.00539435

Table S4: List of annotations of human CK1s substrates enrichment map (Figure 4)

Gene set name	Gene set description	fdr_qvalue
Phosphate metabolic process		
GO:0046031	ADP metabolic process	0.003179664
GO:0006757	ATP generation from ADP	0.001347098
GO:0046034	ATP metabolic process	0.001144815
GO:0019674	NAD metabolic process	5.75E-08
GO:0006734	NADH metabolic process	1.35E-07
GO:0006735	NADH regeneration	6.02E-07
GO:0061621	canonical glycolysis	6.02E-07
GO:0019752	carboxylic acid metabolic process	0.010299148
GO:0006007	glucose catabolic process	2.29E-05
GO:0061718	glucose catabolic process to pyruvate	6.02E-07
GO:0006096	glycolytic process	0.001203496
GO:0061615	glycolytic process through fructose-6-phosphate	2.41E-06
GO:0061620	glycolytic process through glucose-6-phosphate	1.55E-06
GO:0019320	hexose catabolic process	3.81E-04
GO:0046365	monosaccharide catabolic process	0.001180593
GO:0009132	nucleoside diphosphate metabolic process	1.46E-04
GO:0006165	nucleoside diphosphate phosphorylation	3.09E-04
GO:0006753	nucleoside phosphate metabolic process	0.040055485
GO:0009117	nucleotide metabolic process	0.02558125
GO:0046939	nucleotide phosphorylation	3.84E-04
GO:0006082	organic acid metabolic process	0.028097385
GO:0043436	oxoacid metabolic process	0.02517777
GO:0006796	phosphate-containing compound metabolic process	0.00393743
GO:0006793	phosphorus metabolic process	0.003626999
GO:0016310	phosphorylation	0.0231401
GO:0009135	purine nucleoside diphosphate metabolic process	4.27E-04
GO:0009179	purine ribonucleoside diphosphate metabolic process	4.27E-04
GO:0006090	pyruvate metabolic process	1.11E-05
GO:0009185	ribonucleoside diphosphate metabolic process	1.06E-04
Response to stimulus		
GO:0070887	cellular response to chemical stimulus	3.66E-08
GO:0062197	cellular response to chemical stress	0.003187896
GO:0071345	cellular response to cytokine stimulus	9.40E-09
GO:0036294	cellular response to decreased oxygen levels	0.025069536
GO:0034605	cellular response to heat	9.69E-07
GO:0071456	cellular response to hypoxia	0.047898554
GO:0071349	cellular response to interleukin-12	1.11E-05
GO:0071310	cellular response to organic substance	5.46E-09
GO:0033554	cellular response to stress	3.21E-09
GO:0035967	cellular response to topologically incorrect protein	2.29E-06
GO:0034620	cellular response to unfolded protein	1.68E-07
GO:0019221	cytokine-mediated signaling pathway	0.026799497
GO:0070987	error-free translesion synthesis	0.046126886
GO:0035722	interleukin-12-mediated signaling pathway	6.58E-06
GO:0034975	protein folding in endoplasmic reticulum	7.08E-06
GO:1900034	regulation of cellular response to heat	5.08E-05
GO:0080135	regulation of cellular response to stress	0.006255738
GO:0009628	response to abiotic stimulus	3.22E-07
GO:0042221	response to chemical	1.96E-05
GO:0034097	response to cytokine	1.43E-07
GO:0036293	response to decreased oxygen levels	0.035106134
GO:0042493	response to drug	0.012836346
GO:0034976	response to endoplasmic reticulum stress	2.63E-05
GO:0009408	response to heat	2.44E-05
GO:0070671	response to interleukin-12	1.42E-05
GO:0010033	response to organic substance	1.32E-09
GO:0070482	response to oxygen levels	0.015443382
GO:0006950	response to stress	0.003594638
GO:0009266	response to temperature stimulus	0.002464404
GO:0035966	response to topologically incorrect protein	9.48E-11
GO:0006986	response to unfolded protein	3.57E-12

Table S4: List of annotations of human CK1s substrates enrichment map (Figure 4)

Gene set name	Gene set description	fdr_qvalue
Actin cytoskeleton organization		
GO:0030036	actin cytoskeleton organization	0.008966479
GO:0007015	actin filament organization	0.025911115
GO:0030048	actin filament-based movement	0.033506364
GO:0030029	actin filament-based process	5.49E-04
GO:0022607	cellular component assembly	2.66E-12
GO:0044085	cellular component biogenesis	6.17E-19
GO:0051131	chaperone-mediated protein complex assembly	0.003978168
GO:0051276	chromosome organization	0.015155232
GO:0007010	cytoskeleton organization	4.93E-05
GO:0001732	formation of cytoplasmic translation initiation complex	2.18E-04
GO:0007020	microtubule nucleation	0.007735019
GO:0034622	cellular protein-containing complex assembly	5.55E-12
GO:0006996	organelle organization	4.60E-09
GO:0051130	positive regulation of cellular component organization	0.008053748
GO:0098974	postsynaptic actin cytoskeleton organization	0.022789563
GO:0051258	protein polymerization	0.002175293
GO:0065003	protein-containing complex assembly	1.86E-15
GO:0043933	protein-containing complex subunit organization	4.97E-13
GO:0051493	regulation of cytoskeleton organization	0.007759528
GO:0010638	positive regulation of organelle organization	0.002753984
GO:0070507	regulation of microtubule cytoskeleton organization	0.006876633
GO:0031113	regulation of microtubule polymerization	0.046946458
GO:0033043	regulation of organelle organization	1.98E-05
GO:0032271	regulation of protein polymerization	2.92E-04
GO:1902903	regulation of supramolecular fiber organization	0.00203318
GO:0043254	regulation of protein-containing complex assembly	2.21E-04
GO:0022618	ribonucleoprotein complex assembly	2.52E-07
GO:0034063	stress granule assembly	0.009955833
GO:0071826	ribonucleoprotein complex subunit organization	9.65E-08
GO:0097435	supramolecular fiber organization	1.93E-06
Cellular localization & transport		
GO:0006405	RNA export from nucleus	0.023499596
GO:0006403	RNA localization	1.75E-05
GO:0006886	intracellular protein transport	7.22E-10
GO:0046907	intracellular transport	2.06E-17
GO:0051668	localization within membrane	2.04E-05
GO:0051028	mRNA transport	0.033506364
GO:0033036	macromolecule localization	2.40E-10
GO:0050658	RNA transport	0.00539435
GO:0051641	cellular localization	1.66E-16
GO:0071705	nitrogen compound transport	4.32E-10
GO:0070727	cellular macromolecule localization	8.92E-10
GO:0050657	nucleic acid transport	0.00539435
GO:0034613	cellular protein localization	5.54E-10
GO:0051649	establishment of localization in cell	1.73E-19
GO:0045184	establishment of protein localization	3.05E-09
GO:0006913	nucleocytoplasmic transport	4.72E-06
GO:0090150	establishment of protein localization to membrane	1.06E-08
GO:0090151	establishment of protein localization to mitochondrial membrane	0.029794949
GO:0072655	establishment of protein localization to mitochondrion	0.018810777
GO:0072594	establishment of protein localization to organelle	2.93E-16
GO:0051236	establishment of RNA localization	0.006811783
GO:0051168	nuclear export	2.61E-06
GO:0051169	nuclear transport	5.82E-06
GO:0071702	organic substance transport	6.85E-07

Table S4: List of annotations of human CK1s substrates enrichment map (Figure 4)

Gene set name	Gene set description	fdr_qvalue
Cellular localization & transport (end)		
GO:1900740	positive regulation of protein insertion into mitochondrial membrane involved in apoptotic signaling pathway	0.009955833
GO:0051204	protein insertion into mitochondrial membrane	0.012959673
GO:0001844	protein insertion into mitochondrial membrane involved in apoptotic signaling pathway	0.028082124
GO:0008104	protein localization	4.36E-10
GO:0006605	protein targeting	4.16E-10
GO:0015031	protein transport	6.41E-09
GO:0006612	protein targeting to membrane	9.87E-08
GO:0072657	protein localization to membrane	1.83E-06
GO:0070585	protein localization to mitochondrion	0.026281564
GO:0060341	regulation of cellular localization	9.58E-04
GO:1903827	regulation of cellular protein localization	0.001260731
GO:0032880	regulation of protein localization	0.033369095
GO:0033365	protein localization to organelle	1.72E-14
GO:1900739	regulation of protein insertion into mitochondrial membrane involved in apoptotic signaling pathway	0.009955833
GO:0032386	regulation of intracellular transport	0.046112704

Table S5: List of annotations of proteins targeted by L-CK1.2 and phosphorylated in SARS-CoV2 infection.

RNA splicing & processing		
**Gene set name**	**Gene set description**	**fdr-qvalue**
GO:0008380	RNA splicing	7.56E-06
GO:1903311	regulation of mRNA metabolic process	0.002061234
GO:0000398	mRNA splicing, via spliceosome	2.89E-04
GO:0090304	nucleic acid metabolic process	0.023112918
GO:0031123	RNA 3'-end processing	0.021130162
GO:0016071	mRNA metabolic process	1.13E-06
GO:0000375	RNA splicing, via transesterification reactions	3.16E-04
GO:0006397	mRNA processing	9.25E-07
GO:0050684	regulation of mRNA processing	0.001816144
GO:0031124	mRNA 3'-end processing	0.024329926
GO:0006396	RNA processing	1.05E-06
GO:0000377	RNA splicing, via transesterification reactions with bulged adenosine as nucleophile	2.89E-04
GO:0016070	RNA metabolic process	0.009712339

Table S6: List of annotations of proteins targeted by L-CK1.2 and phosphorylated in SARS-CoV2 infection.

Cellular localisation & transport		
**Gene set name**	**Gene set description**	**fdr-qvalue**
GO:0070727	cellular macromolecule localization	0.033862353
GO:0034613	cellular protein localization	0.03115501
GO:1903827	regulation of cellular protein localization	0.034549815
GO:0046907	intracellular transport	0.01168741
GO:0051168	nuclear export	0.0122513
GO:0051169	nuclear transport	2.56E-04
GO:0006913	nucleocytoplasmic transport	0.014728889
